# Supplementary material for: Effects of Different Blanching Methods on the Quality of Tremella fuciformis and Its Moisture Migration Characteristics
Source: Foods. 2023 Apr 17;12(8):1669. doi: 10.3390/foods12081669 (PMC10137464; doi:10.3390/foods12081669)
Supplement: Supplementary file 1 [file foods-12-01669-s001.zip › foods-2301313-supplementary.pdf]

Table S1 The scoring criteria of sensory evaluation of *T. fuciformis*.

Parameter	Description of attributes	Score
Color	Silvery white.	16 ~ 20
	White.	11 ~ 15
	White with blue.	6 ~ 10
	White with yellow.	1 ~ 5
Texture	Soft and plump, tender and elastic.	16 ~ 20
	Excessive hardness, chewy.	11 ~ 15
	Excessive softness, no springiness.	6 ~ 10
	Gummy and viscous.	1 ~ 5
Aroma	Intense characteristic fragrance.	16 ~ 20
	Faint fragrance.	11 ~ 15
	None fragrance.	6 ~ 10
	Some awful odor.	1 ~ 5
Appearance	Opaque and shiny.	16 ~ 20
	Opaque and slightly shiny.	11 ~ 15
	Translucent.	6 ~ 10
	Translucent and lustreless.	1 ~ 5
Flavor	Sapid.	16 ~ 20
	Bland.	11 ~ 15
	Flat.	6 ~ 10
	Insipid.	1 ~ 5
